# Clinical outcomes for 61 cases of hypopharyngeal cancer with synchronous esophageal cancer

**DOI:** 10.1093/jrr/rrz042

**Published:** 2019-06-28

**Authors:** Shixuan Yang, Shuang Yang, Wenjun Liao, Rui Huang, Baisen Li, Shun Lu, Chao Li, Zhaohui Wang, Chunhua Li, Jiao Pei, Hao Wen, Shichuan Zhang

**Affiliations:** 1 Department of Oncology, Affiliated Hospital of Southwest Medical University, No. 25, Taiping street, Luzhou city, Sichuan, China; 2 Department of Oncology, People’s Hospital of Cangxi County, Guangyuan, China; 3 Department of Radiation Oncology, Sichuan Cancer Hospital & Institute, Sichuan Cancer Center, School of Medicine, University of Electronic Science and Technology of China, Chengdu, China; 4 Department of Head Neck Oncology, Sichuan Cancer Hospital & Institute, Sichuan Cancer Center, School of Medicine, University of Electronic Science and Technology of China, Chengdu, China; 5 Institute of Drug Clinical Trial, Sichuan Cancer Hospital and Institute, Sichuan Cancer Center, School of Medicine, University of Electronic Science and Technology of China, Chengdu, China

**Keywords:** hypopharyngeal neoplasms, esophageal neoplasms, chemoradiotherapy, surgery, survival rate

## Abstract

The aim of this research was to provide data from a single-center study of the treatment of synchronous hypopharyngeal cancer (HPC) and esophageal cancer (EC) with different treatment modalities. A total of 61 patients with synchronous HPC and EC were included in this study. Patients were treated with radiotherapy/chemoradiotherapy (28 cases), surgery (9 cases), palliative radiotherapy and/or chemotherapy (17 cases), or supportive care (7 cases). The median radiotherapy doses for EC and HPC in the radiotherapy/chemoradiotherapy group were 64.5 Gy (range, 0–70) and 70 Gy (range, 60–75.2), respectively. Seven patients in the surgery group received pharyngoesophagectomy with gastric pull-up reconstruction, and two received esophagectomy followed by radiotherapy at the hypopharynx. Cox proportional hazard analysis revealed that the outcome of active treatments, including surgery and radiotherapy/chemoradiotherapy, was better than that of conservative care. In survival analysis, patients in the surgery group tended to have a better 3-year overall survival rate than those in the radiotherapy/chemoradiotherapy group (55.6% vs 30.9%); however, this difference was not statistically different (*P* = 0.493). The two groups had similar 3-year progression-free survival rates (30.6% and 33.3%, *P* = 0.420). The current study suggested that radiotherapy/chemoradiotherapy should be considered as an important treatment modality in addition to surgery for synchronous HPC and EC.

## INTRODUCTION

Hypopharyngeal cancer (HPC) and esophageal cancer (EC) are frequently observed as combined malignancies in the upper digestive tract. In a study that included 368 Japanese patients with EC, 41 had HPC either synchronously or metachronously [1]. Wang *et al.* performed esophageal endoscopic screening for 139 HPC patients in Taiwan and confirmed that 40 patients (28.8%) had synchronous esophageal lesions (high-grade dysplasia and invasive tumor) [2]. EC with HPC is not rare in Caucasian patients, but may not occur as frequently in patients living in Eastern Asia [3].

Patients with HPC and EC have poor prognoses. The 5-year overall survival (OS) is ~40% for HPC [4] and 18% for EC [5]. When both diseases are present in the same patient, the prognosis is even poorer. Thus, every patient with HPC–EC double malignancies, especially synchronous diseases, poses a great challenge to clinicians. Thus far, surgery-based treatment has been the dominant treatment strategy in most reported studies, and the role of radiotherapy/chemoradiotherapy in HPC–EC is not well documented in the literature [6]. In the current study, we retrospectively analyzed the treatment results of 61 cases of synchronous HPC–EC in our center and compared the efficacy of different treatment approaches.

## MATERIALS AND METHODS

### Patients

All patients were diagnosed and treated between February 2010 and December 2016 in Sichuan Cancer Hospital. The Warren rules were used to define HPC–EC double malignancies [7]. Briefly, both EC and HPC should have a histopathological diagnosis; they are not contiguous lesions with normal mucosa between two sites. Only secondary tumors diagnosed within 6 months after the initial diagnosis were recognized as synchronous tumors. All non-squamous cell carcinomas, including adenocarcinoma and small cell carcinoma, were excluded. All patients were restaged according to the 7th TNM classification, by Z.S. and W.H. through careful reviewing of pre-treatment CT, barium swallow, and endoscopy.

### Treatment

The treatment decision for any given patient was decided after a discussion between the surgeon and radiation oncologist, and both the patient and patient’s family’s wishes were also respected.

For radiotherapy, the intensity-modulated radiation therapy (IMRT) technique was used. All IMRT fields covered the tumor and positive lymph nodes. Clinical target volumes (CTVs) for the cervix and mediastinum were also included, with volume adjustment according to tumor stage and location. However, only patients who were treated with doses of >40 Gy for EC (not including T0 disease) and >60 Gy for HPC were categorized into the radiotherapy group. For surgery, gastric pull-up (GPU) after pharyngo-laryngo-esophagectomy (PLE) was the major approach. Patients who received esophagectomy combined with radiation for HPC were also categorized into the surgery group. Patients who received radiation doses of ≤40 Gy for EC and ≤60 Gy for HPC and those who received systemic chemotherapy were grouped as having received palliative radiotherapy and/or chemotherapy. The chemotherapy regimens included 5-fluorouracil plus cisplatin/nedaplatin, paclitaxel plus cisplatin/nedaplatin, docetaxel plus 5-fluorouracil plus cisplatin, and single agent chemotherapy (cisplatin or S-1). At least 1 cycle of chemotherapy was administered to patients who received chemotherapy.

### Variables

In addition to clinical stage, body mass index (BMI), liver function, performance status score, and tobacco and alcohol consumption were also included as variables that might influence survival. In our center, we defined abnormal liver function as levels of any one of the following enzymes exceeding the normal range: ALT (alanine aminotransferase), AST (aspartate aminotransferase) and GGT (gamma-glutamyl transpeptidase) or bilirubin. The evaluation of tobacco and alcohol consumption was based on medical records at admission. Tobacco and alcohol consumption were determined by the number of packs smoked per day and grams consumed per day, respectively, multiplied by the number of years of consumption.

### End points

We defined OS as the time interval between the initial diagnosis of either EC or HPC and patient death or last follow-up time, and progression-free survival (PFS) as the time from first diagnosis to disease progression of either EC or HPC and patient death or last follow-up time.

### Statistical analysis

The Cox proportional hazards model was used in the univariate and multivariate analyses to identify prognostic factors. The Kaplan–Meier method was used to generate survival curves, and the log-rank test was used to compare the survival between two groups. The cut-off used to stratify constant variables was determined via an online cut-off calculation module as reported previously [8].

## RESULTS

### Patients’ characteristics

A total of 86 cases of HPC–EC double malignancies were found in the patient database and 67 cases of synchronous diseases were identified. Six patients had inadequate records, so 61 cases were eventually included in this study. The patients’ characteristics are summarized in Table 1. Only 1 female patient was included. The median age was 58 (range, 45–75). A total of 55 patients were diagnosed as having EC combined with HPC at admission, whereas 6 patients had a median interval of 2.6 months between the diagnosis of the two diseases (range, 1–4 months).

**Table 1.** Patients’ characteristics

Variable	Number of patients (%)				
	Total (*n* = 61)	Radiotherapy group (*n* = 28)	Surgery group (*n* = 9)	Palliative radiotherapy and/or chemotherapy group (*n* = 17)	Supportive care group (*n* = 7)
Age					
Median	58	56	62	61	56
Range	45–75	45–75	48–69	46–70	49–69
Gender					
Male	60 (98.36%)	28 (100%)	8 (88.89%)	17 (100%)	7 (100%)
Female	1 (1.64%)	0 (0%)	1 (11.11%)	0 (0%)	0 (0%)
Performance status					
0–1	56 (91.80%)	27 (96.43%)	9 (100%)	13 (76.47%)	7 (100%)
2	4 (6.56%)	1 (3.57%)	0 (0%)	3 (17.65%)	0 (0%)
3	1 (1.64%)	0 (0%)	0 (0%)	1 (5.88%)	0 (0%)
BMI					
Median	20.94	21.18	21.48	20.57	20.42
Range	15.23–27.55	15.89–27.55	18.43–25.91	15.23–24.61	17.21–24.17
Smoking index					
Median	22	23.75	30	10	30
Range	0–80	0–80	0–50	0–80	1–80
Drinking index					
Median	7500	7500	5000	5000	7500
Range	0–20 000	0–20 000	0–15 000	0–20 000	0–15 000
Location of HPC					
Pyriform sinus	49 (80.33%)	24 (85.71%)	6 (66.67%)	14 (82.35%)	5 (71.43%)
Postpharyngeal wall	6 (9.84%)	3 (10.71%)	0 (0%)	2 (11.76%)	1 (14.29%)
Postcricoid area	6 (9.84%)	1 (3.57%)	3 (33.33%)	1 (5.88%)	1 (14.29%)
Location of EC^*^					
Cervical	12 (19.67%)	8 (28.57%)	2 (22.22%)	1 (5.88%)	1 (14.29%)
Upper thoracic	15 (24.59%)	8 (28.57%)	1 (11.11%)	5 (29.41%)	1 (14.29%)
Middle thoracic	26 (42.62%)	10 (35.71%)	3 (33.33%)	10 (58.82%)	3 (42.86%)
Lower thoracic	21 (34.43%)	10 (35.71%)	4 (44.44%)	4 (23.53%)	3 (42.86%)
Stage of HPC					
I	10 (16.39%)	3 (10.71%)	4 (44.44%)	1 (5.88%)	2 (28.57%)
II	6 (9.84%)	5 (17.86%)	1 (11.11%)	0 (0%)	0 (0%)
III	11 (18.03%)	6 (21.43%)	0 (0%)	3 (17.65%)	2 (28.57%)
IV	34 (55.74%)	14 (50.00%)	4 (44.44%)	13 (76.47%)	3 (42.86%)
Stage of EC					
0	4 (6.56%)	2 (7.14%)	1 (11.11%)	1 (5.88%)	0 (0%)
I	17 (27.87%)	7 (25.00%)	3 (33.33%)	5 (29.41%)	2 (28.57%)
II	25 (40.98%)	14 (50.00%)	4 (44.44%)	5 (29.41%)	2 (28.57%)
III	15 (24.59%)	5 (17.86%)	1 (11.11%)	6 (35.29%)	3 (42.86%)

BMI = body mass index, HPC = hypopharyngeal cancer, EC = esophageal cancer. *13 patients had multiple EC lesions, and each lesion was calculated separately.

We mapped the location of the EC and found that 26 cases (42.6%) occurred in the middle thoracic esophagus, whereas 15 cases (24.6%), 21 cases (34.4%) and 12 cases (19.7%) occurred in the upper thoracic, lower thoracic and cervical esophagus, respectively. Only 4 cases were carcinoma *in situ*, and 13 cases had multiple esophageal lesions.

Among the patients, 73.8% of HPC cases and 24.6% of EC cases were at advanced stages. We defined Type A patients as those with early-stage (0–II) EC with early-stage (I–II) HPC, Type B as early EC with advanced-stage (III–IV) HPC, Type C as advanced-stage (III) EC with early-stage HPC, and Type D as having both advanced-stage EC and HPC. There were 12, 34, 4 and 11 cases of Type A, B, C and D patients, respectively.

Most of the patients (51 in 61) had a history of drinking. The median drinking index was 7500. Twenty patients had a drinking index >7500 (range, 8000–20 000). The liver function of these patients was not significantly worse than that of the others (*P* = 0.242). A total of 27 patients had abnormal liver function, and the intensity of chemotherapy (cycles of chemotherapy) for these patients was similar to those with normal liver function (*P* = 0.120).

### Treatment groups

A total of 28 patients received radiation-based treatment. Except for 4 patients with early-stage EC tumors (2 patients with T1 EC received endoscopic therapy at the esophagus plus chemoradiotherapy at the pharynx, and 2 patients with Tis EC received systemic chemotherapy plus chemoradiotherapy at the pharynx), all other patients received radiation at both the EC and HPC sites. Among them, 16 cases received EC and HPC radiotherapy simultaneously, and 8 cases were treated sequentially, with a median interval of 2.75 months (range, 2–4 months). Basically, patients with lower thoracic EC were submitted to sequential radiotherapy, whereas those with middle, upper and cervical EC were treated with one IMRT planning. A 1–1.5 cm gap was added between two plannings for sequential radiotherapy to avoid dose overlapping. The median dose was 64.5 Gy (range, 0–70) for EC and 70 Gy (range, 60–75.2) for HPC.

In total, 17 patients received neoadjuvant chemotherapy, 9 patients received concurrent chemotherapy and/or adjuvant chemotherapy, and another 2 patients received radiotherapy only. The surgery group contained 9 patients. Seven cases received GPU after PLE, 4 patients received neoadjuvant chemotherapy, and 2 received esophagectomy followed by radiotherapy at the hypopharynx.

The palliative radiotherapy and/or chemotherapy group contained 9 patients who received ≤40 Gy for radiotherapy and 8 patients who received chemotherapy as their sole treatment. Seven patients adopted supportive care without any anti-cancer treatment. The clinical characteristics of patients in each treatment group are presented in Table 1.

**Fig. 1.**
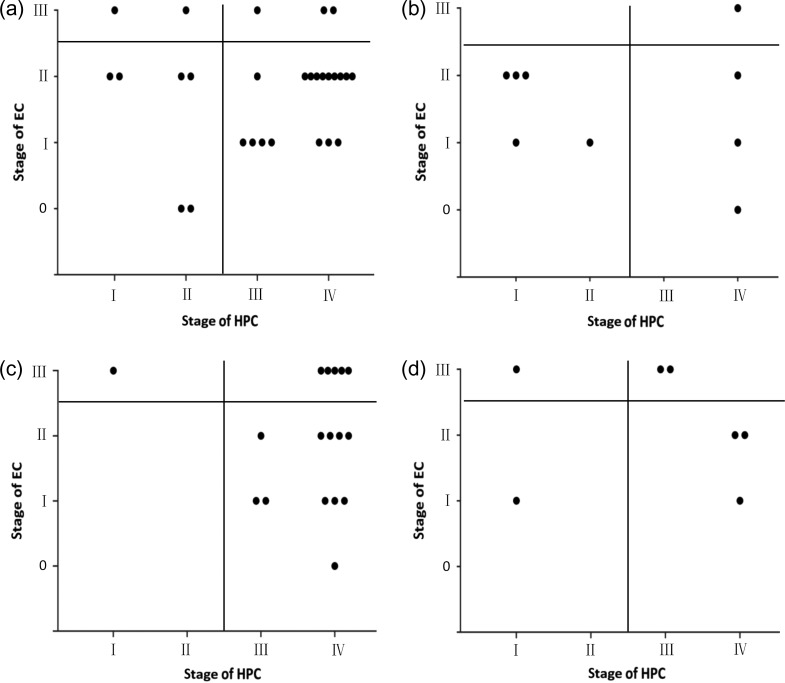
Combined stages of esophageal cancer (EC) and hypopharyngeal cancer (HPC) in different treatment groups. Dots plotted in the lower left quadrant, lower right quadrant, upper left quadrant and upper right quadrant represent type A, B, C and D patients, respectively. There was no significant difference in the distribution of patient types between the radiotherapy/chemoradiotherapy and supportive care groups (*P* = 0.504, rank-sum test). The palliative treatment group had more patients with advanced disease than the radiotherapy/chemoradiotherapy group (*P* = 0.025, rank-sum test). The surgery group contained more type A patients than the radiotherapy/chemoradiotherapy group (55.56% vs 21.43%), but the difference was not statistically significant (*P* = 0.154, rank-sum test). (a) Radiotherapy/chemoradiotherapy group; (b) surgery group; (c) palliative radiotherapy and/or chemotherapy group; and (d) supportive care group.

The number of patients with each of the different combinations of stages of EC and HPC (Types A–D) are shown in Fig. 1. There was no significant difference in the distribution of patient types between the radiotherapy/chemoradiotherapy and supportive care groups (*P* = 0.504, rank-sum test). In contrast, the palliative treatment group had more patients with advanced disease than the radiotherapy/chemoradiotherapy group (*P* = 0.025, rank-sum test), and more patients with early-stage disease (Type A) were seen in the surgery group than in the radiotherapy group (55.56% vs 21.43%), although this was not statistically significant (*P* = 0.154, rank-sum test).

### Treatment outcomes

The median follow-up time was 39 months (range, 11–61 months). A total of 44 (72.1%) patients died due to progression of either EC or HPC. Only 17 patients were alive at their last follow-up time; among them, 10 patients were disease-free. The median survival for the whole cohort, the radiotherapy group, surgery group, palliative radiotherapy and/or chemotherapy group, and supportive care group was 18, 24, 39, 11 and 5 months, respectively.

### Univariate and multivariate analysis

**Fig. 2.**
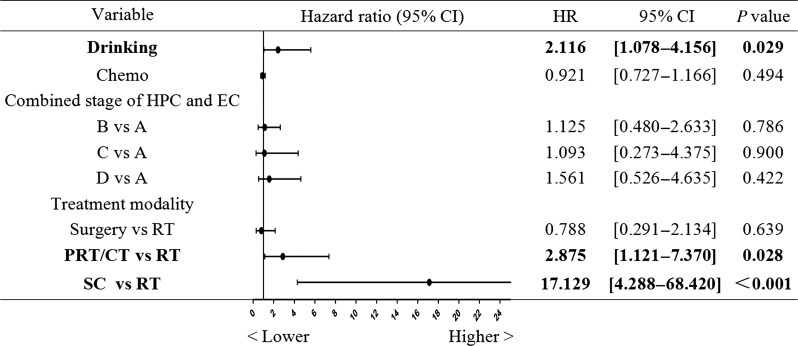
Multivariate analysis for factors associated with OS. Bold writing indicates results with *P* < 0.05. HR = hazard ratio, CI = confidence interval, Chemo = cycles of double drug chemotherapy, HPC = hypopharyngeal cancer, EC = esophageal cancer, RT = radiation therapy, PRT/CT = palliative radiotherapy and/or chemotherapy, SC = supportive care.

To elucidate the factors associated with OS, we included treatment modality, age, BMI, liver function, performance status score, drinking index, smoking index, stage, and number of cycles of chemotherapy in the univariate analysis. The results showed that drinking index (*P* = 0.033) and cycles of double-drug chemotherapy (*P* = 0.002) had impacts on the OS (Table 2). In addition, combined stage Type D patients were associated with worse prognosis than Type A patients [hazard ratio (HR) 3.485, *P* = 0.009]. For treatment modality, patients in the surgery group tended to have better outcomes than those in the radiotherapy group (HR 0.768, *P* = 0.585), whereas patients of the other two groups had worse prognosis than those of the radiotherapy group (*P* < 0.001 for both palliative treatment and supportive care groups). We then included these four variables in a multivariate analysis. The initial results showed that only treatment modality and drinking index had a significant influence on OS. We then performed a group comparison within these two variables. For treatment modality, the radiotherapy group was used as a reference. For drinking index, the patients were divided into two groups according to a cut-off value (7750) determined by an online cut-off calculation module, as described in the Materials and Methods section [8]. The results showed that surgery-based therapy tended to yield better outcome than radiotherapy-based treatment. However, this difference did not meet the *P* < 0.05 significance threshold [HR 0.788, 95% confidence interval (CI) = 0.291–2.134, *P* = 0.639, Fig. 2]. The patients in the palliative radiotherapy and/or chemotherapy group and supportive care group had significantly worse results than those in the radiotherapy group (HR 2.875, 95% CI = 1.121–7.370, *P* = 0.028; HR 17.129, 95% CI = 4.288–68.420, *P* < 0.001, respectively). The results also showed that patients who were heavy drinkers (>7750) had a significantly poorer prognosis than their counterparts (HR 2.116, 95% CI = 1.078–4.156, *P* = 0.029).

**Table 2.** Univariate analysis for factors associated with OS

Variable	HR	95% CI	*P* value
Age	1.015	0.976–1.057	0.453
Performance status	1.583	0.855–2.928	0.144
BMI	0.980	0.870–1.104	0.742
Liver function	1.129	0.617–2.065	0.694
Smoking	1.013	0.999–1.027	0.076
Drinking	1.948	1.054–3.601	0.033
Stage of HPC	1.129	0.872–1.464	0.358
Stage of EC	1.133	0.786–1.631	0.503
Chemo	0.746	0.619–0.900	0.002
Combined stage of HPC and EC			0.044
B vs A	1.447	0.641–3.266	0.374
C vs A	1.575	0.417–5.951	0.503
D vs A	3.485	1.373–8.846	0.009
Treatment modality			<0.001
Surgery vs RT	0.768	0.297–1.983	0.585
PRT/CT vs RT	3.794	1.833–7.851	<0.001
SC vs RT	19.771	6.361–61.455	<0.001

HR = hazard ratio, CI = confidence interval, BMI = body mass index, HPC = hypopharyngeal cancer, EC = esophageal cancer, Chemo = cycles of double drugs chemotherapy.

### Survival analysis

**Fig. 3.**
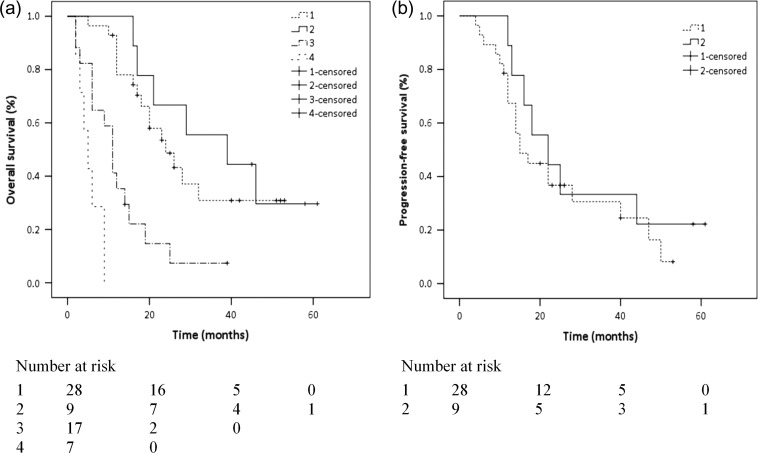
Kaplan–Meier survival curves. Groups are numbered as: 1, radiotherapy/chemoradiotherapy; 2, surgery; 3, palliative radiotherapy and/or chemotherapy; and 4, supportive care. (a) Overall survival. There was no difference between the radiotherapy/chemoradiotherapy and surgery groups (*P* = 0.493), whereas the patients in the palliative radiotherapy and/or chemotherapy and supportive care groups had significantly worse survival rates than those in the radiotherapy/chemoradiotherapy group (*P* < 0.001 for both). (b) Progression-free survival curves of the patients in the radiotherapy/chemoradiotherapy and surgery groups. There was no significant difference between the two curves (*P* = 0.420).

To further investigate the impact of treatment choices and alcohol consumption on disease prognosis, we used the Kaplan–Meier method to compare the survival of patients in different groups (Fig. 3). Similar to the multivariate analysis, the OS curves for the surgery and radiotherapy groups were well separated from those of the other two groups. The 3-year OS rate for the whole cohort, radiotherapy/chemoradiotherapy group and surgery group was 25.5%, 30.9% and 55.6%, respectively. The 3-year PFS rate for the radiotherapy/chemoradiotherapy group and surgery group was 30.6% and 33.3%, respectively. Patients in the surgery group had relatively better overall survival than those in the radiotherapy group, although the log-rank test suggested that there was no significant difference between them, either for OS or PFS (*P* = 0.493 and *P* = 0.420, respectively). Heavy drinking patients also had poorer survival compared with non-heavy drinking patients (*P* = 0.028), proving that heavy alcohol consumption is an independent factor that exerts a harmful influence on patient outcome.

## DISCUSSION

Although synchronous HPC–EC is frequently seen in the clinics and is difficult to handle, its optimal treatment strategy has not been well discussed. For patients with resectable disease, PLE followed by GPU was reported as a mainstay treatment in most studies so far. This technique was introduced in 1960 for treating cervical EC and late-stage HPC [9] and was well known for its high post-operative morbidity and mortality in the early years. With the evolution of surgical procedures and perioperative care, the treatment outcome has significantly improved. Butskiy *et al.* reviewed 77 studies published between 1960 and 2014 and found that mortality was decreased from 29% (1966–1979) to 4% (1995–2008) [6]. However, anastomotic complications, such as leakage and fistula formation, were still observed at a frequency of 8–47% in recent studies (from 2013 to 2014). Other complications, including conduit stricture, pulmonary infection, and wound infection, were also reported [10]. Despite aggressive surgery, the prognosis of patients with synchronous HPC–EC remains poor, with an estimated 5-year survival of 7–24.2% [11, 12].

Although radiotherapy/chemoradiotherapy has been recommended as the first-line treatment for both advanced HPC and EC, the role of radiotherapy/chemoradiotherapy in treating synchronous HPC–EC has not been well-established. Two studies reported the treatment results of chemoradiotherapy on synchronous EC and head and neck (HN) cancer, most of which were HPC [13, 14]. In an earlier study published in 2011, 33 patients received HN and mediastinal radiation. The median dose for HN cancer was 70 Gy (range, 60–70.5 Gy) and that for EC was 60 Gy (range, 45–70 Gy). The 2-year OS and PFS was 44% and 33%, respectively. The other study compared the outcomes of chemoradiotherapy on EC with multiple primary cancers and those with single lesions. The 2-year OS and PFS was 52.2% vs 68.9% and 32.9% vs 54.0%, respectively. The results of the later study were superior to those of the earlier study; however, it should be pointed out that most of the patients (43 in 53, 81.8%) in the second study had multiple EC lesions. Only 8 patients had EC with HPC, and 2 had EC with gastric cancer.

To the best of our knowledge, the current study is the first to compare the efficacy of different treatment strategies for synchronous HPC–EC. The 3-year OS for the entire cohort was 25.5%, which was comparable with that reported by previous studies [13]. Multivariate analysis revealed that treatment strategy significantly influences prognosis. Patients who received either surgery-based therapy or radiotherapy/chemoradiotherapy had superior survival to those who received more conservative care. The results also showed that patients in the surgery group tended to have better prognosis than those in the radiotherapy group, suggesting that for patients with resectable disease, surgery should still be considered as the first-line treatment. The efficacy of radiotherapy in treating synchronous HPC–EC was also clearly demonstrated. Due to the limited number of patients with resectable disease in both groups, it is difficult to compare the efficacy of surgery-based and radiotherapy-based treatment in an objective way. However, it is noticeable that although the radiotherapy group contained a lower percentage of patients with early-stage disease (Type A) than the surgery group (21.43% vs 55.56%), the survival rate of the patients in these two groups was quite similar (*P* = 0.493 for OS and *P* = 0.420 for PFS), suggesting that radiotherapy-based treatment should be considered as the first-line treatment for those with unresectable disease, and that for resectable disease it is also a reasonable treatment choice.

Similar to Shinoto’s study [13], for radiotherapy planning in our study, fields included both neck and mediastinum for most patients, and the prescribed doses were usually >60 Gy for EC and >66 Gy for HPC. It is likely that patients can tolerate such curative doses covering large fields, because there was no treatment-related mortality. However, the absence of a toxicity profile, largely due to incomplete descriptions in the medical records, is one of the major limitations of this study. With simultaneous doses of >66 Gy for HPC and >60 Gy for EC (for some patients, it was a 70–66 combination), Grade 3–4 acute mucositis would have occurred in most patients and might have had a negative impact on survival. Dose adjustments in these patients need to be further explored in the future.

The current study also found that heavy alcohol consumption was an independent factor associated with poorer prognosis, suggesting that the treatment decision should take the patient’s drinking index into consideration.

In conclusion, although this retrospective study had a limited number of patients, it provides the first direct comparison of treatment strategies for synchronous HPC–EC. The results demonstrated that active treatments of either PLE–GPU surgery or definitive radiotherapy/chemoradiotherapy increased patient survival compared with conservative care. Importantly, in addition to surgery, radiotherapy/chemotherapy was proved to be an effective treatment for the treatment of synchronous HPC–EC. The results suggested that radiotherapy/chemoradiotherapy should be recommended as the first-line treatment for patients with unresectable disease and could also be considered in addition to surgery for resectable disease. We propose that patients with synchronous HPC–EC should be strongly recommended to attend multidisciplinary team discussions. Treatment decision should be cautiously made based on a full understanding of the advantages and disadvantages of the different treatment approaches.
